# Survival Prediction Model Using Clinico-Pathologic Characteristics for Nonsmall Cell Lung Cancer Patients After Curative Resection

**DOI:** 10.1097/MD.0000000000002013

**Published:** 2015-11-13

**Authors:** Ching-Yang Wu, Jui-Ying Fu, Ching-Feng Wu, Ming-Ju Hsieh, Yun-Hen Liu, Yi-Cheng Wu, Cheng-Ta Yang, Ying-Huang Tsai

**Affiliations:** From the Division of Thoracic and Cardiovascular Surgery, Department of Surgery, Chang Gung Memorial Hospital, Linkou; Chang Gung University (C-YW, C-FW, M-JH, Y-HL, Y-CW); Division of Chest and Critical Care, Department of Internal Medicine, Chang Gung Memorial Hospital, Linkou; Chang Gung University (J-YF); Division of Chest and Critical Care, Department of Internal Medicine, Change Gung Memorial Hospital, Taoyuan; Chang Gung University (C-TY); and Division of Chest and Critical Care, Department of Internal Medicine, Chang Gung Memorial Hospital, Chiayi; Chang Gung University (Y-HT).

## Abstract

The current TNM staging system did not provide disease relapse information. The aim of study was try to establish a predictive survival model for disease and overall survival in nonsmall cell lung cancer patients who presented as resectable disease and to develop a reference for follow-up imaging tool selection.

From January 2005 to December 2011, 442 patients who initially presented as resectable disease (stages I–IIIa) and received anatomic resection and mediastinal lymph node dissection were included in the study.

Medical charts were thoroughly reviewed and clinico-pathologic factors were collected and analyzed.

Visceral pleural invasion, tumor size >5 cm, and postoperative adjuvant therapy were identified as risk factors for poorer disease-free survival. The 5-year disease-free survival from score 0 to 3 was 68.7%, 46.6%, 31.9%, and 26.1%, respectively. The disease relapse percentage for scores 0 to 3 were 26.49%, 50.61%, 65.05%, and 73.81%, respectively. For analysis of overall survival, age >60 years, tumor size >3 cm, and total metastatic lymph node ratio >0.05 were correlated to worse overall survival. Because greater age may be correlated with poor general condition, we re-scored risk factors that correlated to disease severity that ranging from 0 to 2. The 5-year overall survival range from score 0 to 2 was 56.3%, 43.1%, and 13.1%, respectively.

Poor prognostic factors correlated to disease-free survival were tumor size >5 cm, visceral pleural invasion, and patients needing to receive postoperative adjuvant therapy. Disease-free survival of resectable nonsmall cell lung cancer patients and disease relapse can be stratified by these 3 factors. Chest tomography may be recommended for patients with 1 or more poor disease-free survival risk factors.

## INTRODUCTION

Lung cancer is the leading cause of death by malignancy worldwide. The current TNM staging system as proposed by the American Joint Committee on Cancer (AJCC) classifies the disease invasion status by components including tumor characteristics, status of involved lymph nodes, and distant metastasis conditions. The system groups different disease invasion levels into one stage on the basis of similar disease-free and overall survival curves.^1–4^ Therefore, the TNM system can only provide predictive information about disease-free and overall survival for different stages but is considered the most important prognostic factor in surgically resected patients.^5^

Many prognostic factors have been identified in previous literature. From the view of tumor cell metabolic capacity, high standard uptake value (SUV max) of the tumor area may be related to poor survival among stage I to III disease.^6,7^ Among patients who received anatomic resection and were identified as pathologic stage IIIA, pathologic tumor classification and mediastinal lymph node involvement have been identified as independent prognostic factors for disease-free survival.^8^ In addition, patients without lymph node involvement or with lower metastatic lymph node ratio have been correlated to better survival.^9,10^ From the view of microscopic presentation, actual tumor size,^11–14^ visceral pleura,^15–19^ and angiolymphatic invasion status^20–22^ have been correlated to patient's survival. However, these studies have only identified the prognostic factors that correlated to survival for specific stages without further analysis of disease relapse. In addition, the effect of postoperative treatments was not included for analysis. We have had no ideal survival prediction model that could be utilized as survival prediction and reference for selection of follow-up imaging tools. This may explain why the literature reveals no survival difference between different follow-up imaging programs, such as chest tomography (CT) and chest plain film (CXR).^23–25^

In this study, we summarize all clinico-pathologic factors, including patho-histologic characteristics of cell type, lymph node involvement, and postoperative treatment, and analyze the effects on disease and overall survival. We also attempt to further analyze the relationship between predictive survival model and disease relapse. The aim of this study was not only to establish a predictive survival model for disease or overall survival in nonsmall cell lung cancer patients who presented as resectable disease, but also to develop a reference for follow-up imaging tool selection.

## MATERIALS AND METHODS

### Patient Population

From January 2005 to December 2011, 609 lung cancer patients received surgery in Chang Gung Memorial Hospital. Exclusion criteria were as follows: patients who received wedge resection (43 patients), small cell lung cancer patients (11 patients), pathologic report showing positive resection margin confirmed as stage IIIB or IV (25 patients), and patients who received neoadjuvant therapy (88 patients). Thus, only 442 patients who initially presented as resectable disease (stages I–IIIa) and received anatomic resection and mediastinal lymph node dissection were included in the study. Medical charts were thoroughly reviewed and clinico-pathologic factors were collected. A medical ethics review was approved by the ethics committee of Chang Gung Memorial Hospital under the Institutional Review Board number 103-5631B.

### Disease Evaluation

Patients received a chest computed tomography (CT) scan for disease evaluation, including tumor size and location, mediastinal lymphoadenopathy, and possible extrapulmonary lesions existing in the lower neck and upper abdomen. If suspicious pulmonary lesions were found, tissue proofing by bronchoscopy or CT was arranged for diagnosis confirmation. If no definite diagnosis was reached, repeat biopsy or surgical biopsy were performed prior to anatomic resection in the same operation. Distant metastases were ruled out by bone scan, positron emission tomography (PET) or positron emission tomography–computed tomography (PET-CT). In addition, brain CT or magnetic resonance imaging (MRI) were performed in order to exclude the possibility of central nervous system metastases.

Spirometry was arranged in order to identify the pulmonary reserve as a reference for resection range.

### Surgery, Postoperative Therapy, and Surveillance

Anatomic resection was performed for resectable disease via thoracotomy or video-assisted thoracoscopic surgery (VATS). The tumor location, corresponding pulmonary vessels and lobar or main bronchial involvement were first individually identified. These structures were secured with sutures or endoscopic staples and then divided. Complete mediastinal lymph node dissection was done after anatomic resection was completed. The resected specimen was thoroughly examined by pathologists. Postoperative adjuvant therapies were determined according to final pathologic stage. Patients returned to the outpatient department within a 3-month interval. Complete physical examinations were done and general conditions recorded. Chest plain film or chest CT were utilized as surveillance imaging tools depending on physician preference.

### Disease Relapse and Further Management

**FIGURE 1:**
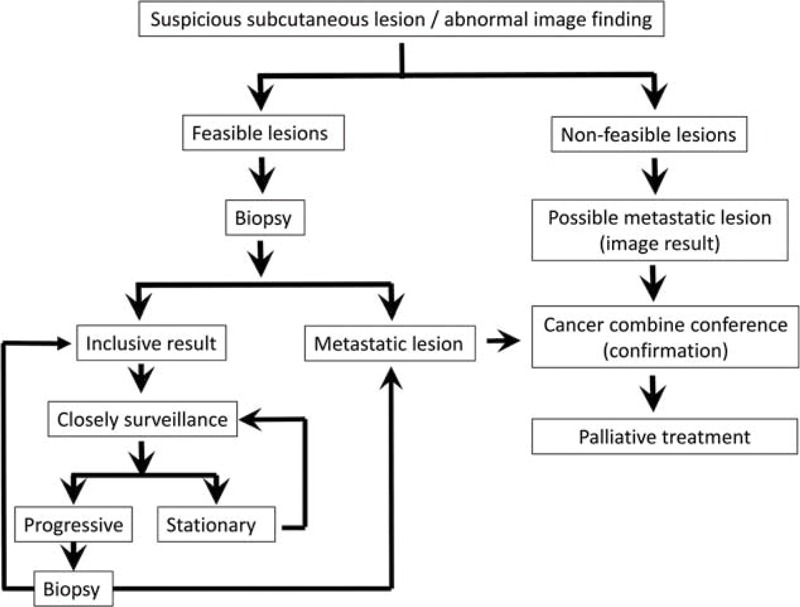
Management algorithm for patients with suspicious metastatic lesions.

If suspicious palpable subcutaneous lesions or abnormal image findings were encountered in the physical examination, further workup was warranted. Tissue proofing for these lesions was mandatory wherever feasible. If biopsy data confirmed metastases correlated to the previous lung cancer, disease relapse was identified. If biopsy result showed negative, close surveillance was recommended at 3-month intervals. If imaging results showed progressive change in the serial follow up, repeat biopsy was warranted. For patients with suspicious lesions where tissue proofing was not feasible, diagnosis confirmation could only reached through extensive discussion. The management algorithm is summarized in Fig. 1.

### Statistics

All collected clinicopathologic factors were evaluated by univariate analysis. Categorical variables were compared using chi-squared or Fisher's exact tests. Disease-free survival was defined as no evidence of relapse in the period from the date of the operation to the last follow-up date. Overall survival was defined as the period between the operation date and death. For patients with feasible lesions, disease relapse date was defined as date of definite pathologic diagnosis. If lesions were not feasible, disease relapse was defined as positive image finding in the surveillance program. Survival status was further analyzed and represented with a Kaplan–Meier curve. A *P* value <0.05 was considered statistically significant. All the analyses were performed using SAS, version 9 (SAS Institute, Cary, NC).

## RESULTS

From January 2005 to December 2011, 442 patients who initially presented as resectable disease (stages I–IIIa) and received anatomic resection and mediastinal lymph node dissection were included into this study. The mean age was 62.58 ± 11.09 years and 234 patients (53.4%) were male. The majority of patients (322/422, 72.8%) were adenocarcinoma. Mean tumor size was 3.25 ± 1.72 centimeters. Under microscopic examination, visceral pleural invasion and angiolymphatic invasion were identified in 49.3% and 33.7% patients, respectively; 59.9% (264/422) of patients received anatomic resection and mediastinal lymph node dissection via thoracotomy. The mean total number of dissected lymph nodes was 18.43 ± 10.67 and the mean total number of metaststic lymph nodes was 1.03 ± 2.46. The final pathologic staging and further postoperative adjuvant therapy are summarized in Table 1. We further analyzed the relationship between survival, including disease-free and overall survival, and these clinicopathologic factors.

**TABLE 1:**
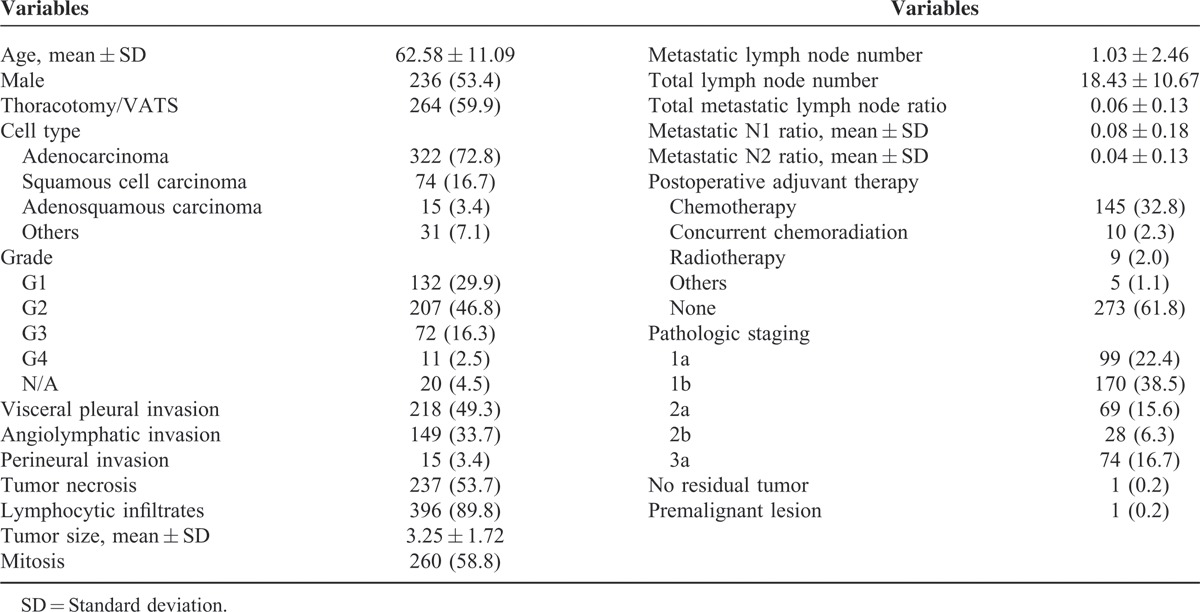
Characteristics

**FIGURE 2:**
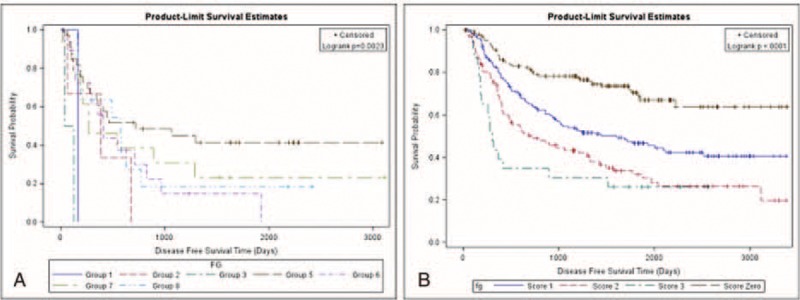
Disease-free survival among stratified subgrouping with risk factors. (A) Disease-free survival curve of patients (8 groups). Group 1: Tumor size >5 cm; visceral pleura invasion (+), with postoperative adjuvant therapy; Group 2: Tumor size >5 cm; visceral pleura invasion (−), with postoperative adjuvant therapy; Group 3: Tumor size >5 cm; visceral pleura invasion (+), without postoperative adjuvant therapy; Group 4: Tumor size >5 cm; visceral pleura invasion (−), without postoperative adjuvant therapy; Group 5: Tumor size ≤5 cm; visceral pleura invasion (+), with postoperative adjuvant therapy; Group 6: Tumor size ≤5 cm; visceral pleura invasion (−), with postoperative adjuvant therapy; Group 7: Tumor size ≤5 cm; visceral pleura invasion (+), without postoperative adjuvant therapy; Group 8: Tumor size ≤5 cm; visceral pleura invasion (−), without postoperative adjuvant therapy. (B) Disease-free survival of patients (4 groups). Score 0: Patients with tumor size ≤5 cm, no visceral pleura invasion and without need of postoperation adjuvant therapy; Score 1: Patients has 1 of following risk factors, such tumor size >5 cm, visceral pleura invasion, the need of postoperative adjuvant therapy; Score 2: Patients has 2 of following risk factors, such tumor size >5 cm, visceral pleura invasion, the need of postoperative adjuvant therapy; Score 3: Patients with tumor size >5 cm, visceral pleura invasion and need postoperative adjuvant therapy.

**FIGURE 3:**
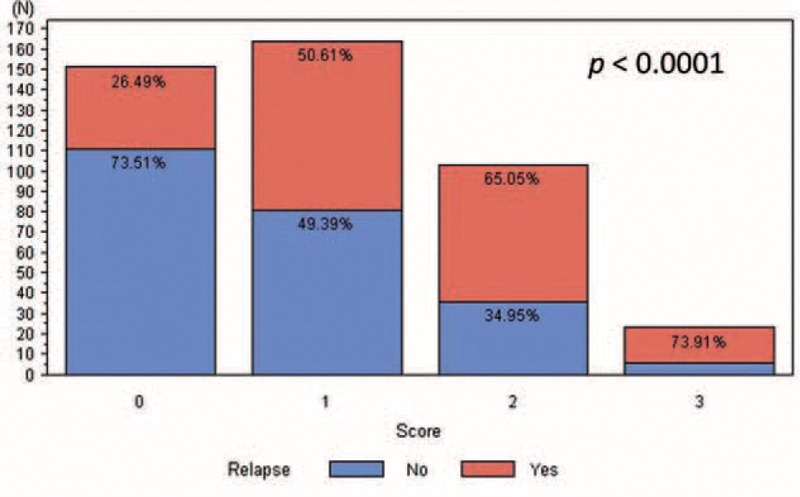
Relapse percentage of each group.

In univariate analysis of disease-free survival, we identified tumor size (*P* < 0.001), visceral pleural invasion (*P* = 0.01), metatstatic N1 ratio (*P* < 0.001), metatstatic N2 ratio (*P* < 0.001), total metatstatic lymph node ratio (*P* < 0.001), thoracotomy (*P* = 0.02), and postoperative adjuvant therapy (*P* < 0.001) as correlated to disease-free survival (Table 2A). In multivariate analysis, only visceral pleural invasion (hazard ratio: 1.52; *P* = 0.002, 95% confidence interval 1.15–2.12), tumor size >5 cm (hazard ratio: 1.46; *P* = 0.05, 95% confidence interval 0.99–2.12), and postoperative adjuvant therapy (hazard ratio: 1.97; *P* < 0.0001, 95% confidence interval 1.49–2.62) were identified as risk factors for poorer disease-free survival (Table 2B). We further stratified all patients with these 3 poor prognostic factors into 8 subgroups and calculated the disease-free survival curve of each subgroup. We identified the patients who, without these 3 poor prognostic factors had the best disease-free survival and those who, with all 3 prognostic factors had the worst disease-free survival (Fig. 2A). In addition, disease-free survival of each subgroup was separated (Fig. 2A, *P* = 0.0023). Since the hazard ratios of the 3 risk factors were similar, we created a simple scoring system. Patients received 1 point for each 1 of these risk factors, and were thus further scored according to their poor prognostic factor number, ranging from 0 to 3. The 5-year disease-free survival from score 0 to 3 was 68.7%, 46.6%, 31.9%, and 26.1%, respectively (Fig. 2B, *P* < 0.001). In addition, for patients with lower score, that is, fewer poor prognostic factors, lower relapse rates were noted. The disease relapse percentage for scores 0 to 3 were 26.49%, 50.61%, 65.05%, and 73.81%, respectively (Fig. 3).

**TABLE 2:**
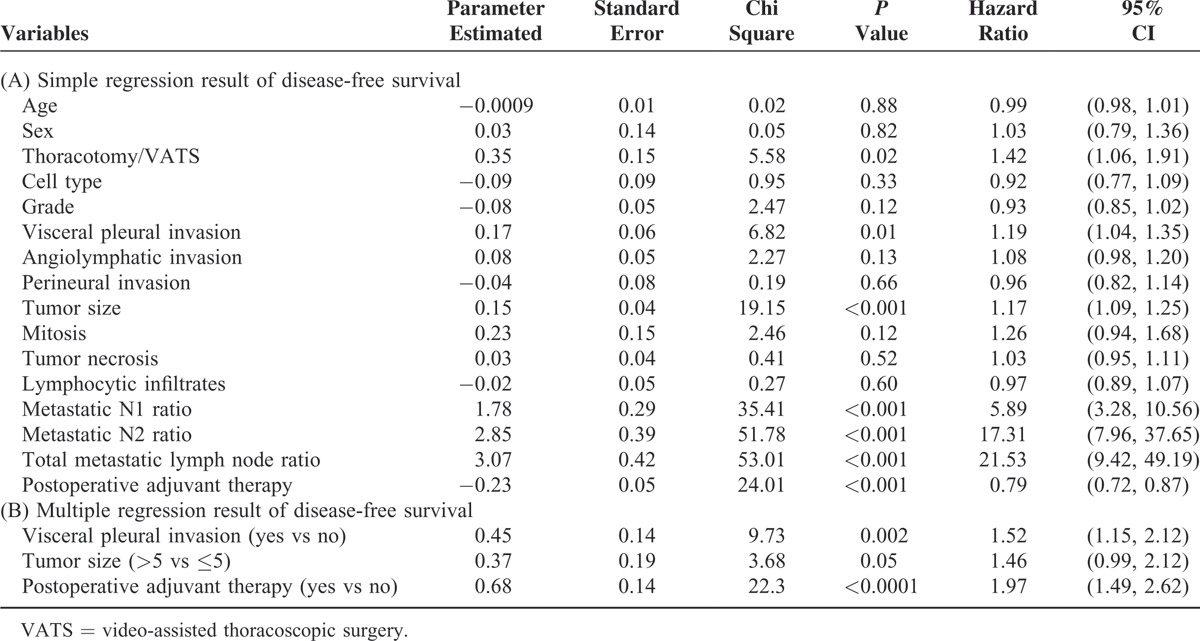
Regression Result of Disease-Free Survival

**FIGURE 4:**
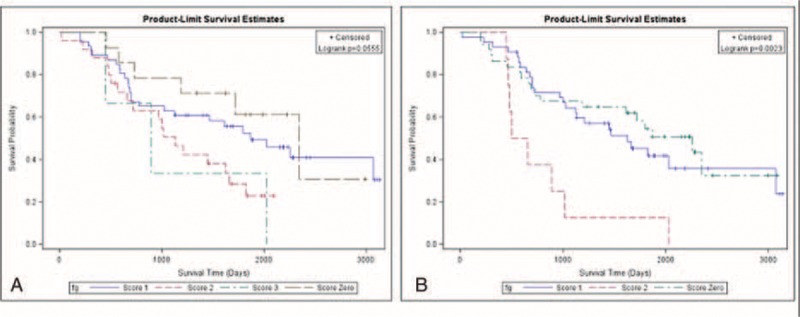
Overall survival among stratified subgrouping with risk factors. (A) Stratified overall survival according to 3 risk factors, including age, tumor size, and total metastatic lymph node ratio (4 groups). (B) Stratified overall survival according to 2 risk factors, including tumor size and total metastatic lymph node ratio (3 groups).

In univariate analysis of overall survival, we identified age (*P* < 0.004), gender (*P* < 0.02), tumor size (*P* = 0.01), mitosis (*P* = 0.04), metastatic N1 ratio (*P* < 0.001), metastatic N2 ratio (*P* < 0.001), total metastatic lymph node ratio (*P* < 0.001), thoracotomy (*P* = 0.02), and postoperative adjuvant therapy (*P* < 0.001) as correlated to overall survival (Table 3A). In multivariate analysis, age >60 years (hazard ratio: 1.45; *P* = 0.02, 95% confidence interval 1.07–1.97), tumor size >3 cm (hazard ratio: 1.41; *P* = 0.03, 95% confidence interval 1.03–1.94), and total metastatic lymph node ratio >0.05 (hazard ratio: 2.28; *P* < 0.001, 95% confidence interval 1.69–3.11) were correlated to worse overall survival (Table 3B). The overall survival of patients with different score is shown in Fig. 4A (*P* = 0.055). Because greater age may be correlated with poor general condition, we re-scored risk factors that correlated to disease severity that ranging from 0 to 2. The 5-year overall survival range from score 2 to 0 was 56.3%, 43.1%, and 13.1%, respectively (Fig. 4B, *P* = 0.0023).

**TABLE 3:**
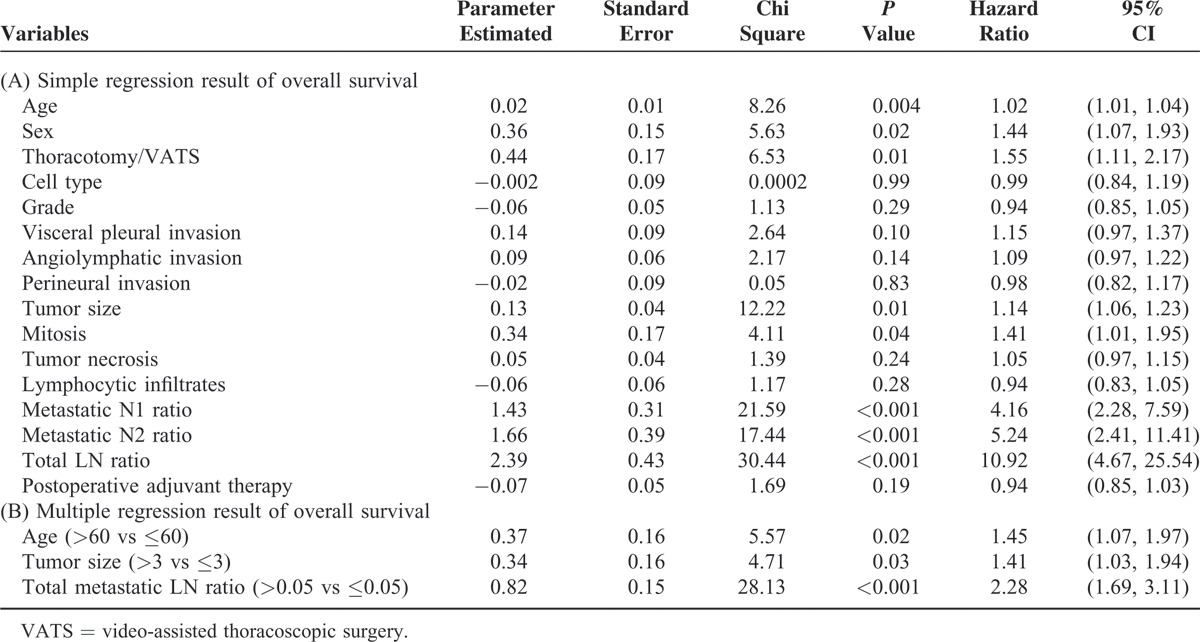
Regression Result of Overall Survival

## DISCUSSION

According to the National Comprehensive Cancer Network (NCCN) guideline, curative-intent resection, that is, anatomic resection and mediastinal lymph node dissection, are recommended for nonsmall cell lung cancer patients who initially present as resectable disease.^26^ Curative-intent tumor resection plays a crucial role in disease-free survival in resectable disease. In this study, we attempt to include all clinicopathologic factors in prognosis analysis. We identified 3 poor prognostic factors correlated to disease-free survival, including tumor size >5 cm, visceral pleural invasion, and patients needing postoperative adjuvant therapy. We were able to utilize these factors to stratify patients into 8 groups with separated cumulative disease-free survival curves (Fig. 2A, *P* = 0.0023). In addition, we devised a simple scoring system that incorporates these 3 poor prognostic factors. Our findings revealed that the score system could differentiate the disease-free survival for patients with different numbers of risk factors (Fig. 2B, *P* < 0.001). Furthermore, we determined the relapse percentage of patients with different numbers of risks factors (Fig. 3). Our result not only provided the same predicting power for disease-free survival as the TNM staging system did but it also correlated to the risk of relapse (Fig. 3). For analysis of overall survival, we identified age >60 years, and disease severity, including tumor size >3 cm and total metastatic lymph node ratio as correlated to patients’ overall survival. Patients who classified with score 1 had better overall survival than those classified as score 2 after 1000 days. In addition, patients who classified as score 0 had better overall survival than those identified as score 3 after 500 days. Because of limited patients were classified as score 0 and 3, the slope of survival curve was declined abruptly and cause interference. The reason that the predicting power of these 3 factors for overall survival only achieved marginal statistical significance (Fig. 4A, *P* = 0.055). Because greater age may be correlated general condition and influenced overall survival, we reanalyzed the relationship between overall survival and factors that correlated to disease severity, and found that patients’ overall survival correlated to the number of risk factors related to disease extension status (Fig. 4B, *P* = 0.0023).

The current TNM staging system groups different tumor invasion conditions presenting with similar disease-free and overall survival into one stage. Therefore, the TNM staging system only provides information about survival but not risk of disease relapse. A survival prediction model recently proposed by Brunelli et al included 3 risk factors for poor prognosis, including age, preoperative carbon monoxide lung diffusion capacity, and preoperative quality-of-life physical commentary score 106.^27^ Although the 5-year overall survival of patients with score 0 was 6 times longer than those with score 3, some limitations remain. This model can only predict overall survival but not disease-free survival and determination of the preoperative quality-of-life physical component summary score is too complicated for extensive clinical use. Furthermore, most risk factor analysis articles focus on early stage patients or specific clinical scenarios and do not document disease relapse.^8–10,28–31^ In these articles, many factors of poor prognosis have been identified, including visceral pleural invasion,^15,17–19,32^ angiolymphatic invasion,^16,20–22,33,34^ tumor size,^12–14,35^ metastatic lymph node number or ratio,^9,10^ and postoperative adjuvant therapy.^36–38^ The prognostic factors that we identified as correlated to disease-free survival and overall survival were similar to those identified in previous studies. However, our study was quite different than previous studies, since we included not only all histopathologic characteristics but also postoperation management factors in survival analysis of nonsmall cell lung cancer patients presenting as resectable disease. Our result identified risk factors that were related to different clinical scenarios which had different clinical significance. In this study, 60.9% of cases were classified as stage I (stage 1A:22.4%; stage IB: 38.5%) resulting in 2 of 3 identified factors being correlated to early stage without lymph node involvement. In addition, postoperative adjuvant therapy was recognized as a prognostic factor that correlated with patients who were classified as stages IIA to IIIA because postoperative adjuvant therapy is recommended according to NCCN guidelines. Furthermore, these risk factors could be used to stratify patients with resectable disease into 4 groups by a simple score system (Fig. 2B, *P* < 0.001). We found that the lower the scores a patient got, the lower the incidence of disease relapse (Fig. 3). Since all clinical components were taken into consideration, our result is well suited for clinical use and overcomes the limitation of previous studies which focused on a specific population. For analysis of overall survival, we only considered that patients’ overall survival was correlated to the number of risk factors related to disease extension status (Fig. 4B, *P* = 0.0023). Although age >60 years was identified as a poor prognostic factor in overall survival, the predictive power of overall survival remains unclear, and may be related to disease evolution of lung cancer and treatment modality. The former refers to the increased percentage of young female patients diagnosed with adenocarcinoma and the latter refers to the use of tyrosine kinase inhibitor. These changes associate patients with relatively younger age with prolonged survival.

The most important clinical significance of our result was to provide a reference for choosing an adequate surveillance imaging tool for follow up. A literature review shows that there is no standard postoperative surveillance program and it varies between institutes and physicians.^23–25^ Chest tomography may be better able to detect early disease relapse than chest plain film.^39^ However, we found no difference in survival between patients followed up with chest tomography and those followed up with chest plain film.^40^ In addition, medical costs for patients with chest tomography were 16.6% higher than without tomography.^41^ Gourcerol et al also demonstrated that an aggressive postoperative surveillance program with acceptable cost may improve patients’ survival.^25^ From the point of view of disease-free survival, our result revealed that patients with more poor prognostic factors, that is, higher scores, would have higher risk of disease relapse. Chest tomography may be recommended for patients with 1 or more poor disease-free survival risk factors, because more than half of these patients were identified with disease relapse. For these high-risk patients, survival could be improved if the relapse lesion is detected earlier. This principle is not only helpful to improve patients’ survival at acceptable cost but also reduces global medical cost.

However, some limitations remain. First, our study was a retrospective study and included all cell types of nonsmall cell lung cancer for analysis. Therefore, we did not further analyze the epidermal growth factor receptor mutation and corresponding tyrosine kinase inhibitor effect. This may be the reason that age >60 years was identified as a poor prognostic factor in overall survival, but the predictive power of overall survival remained unclarified. Second, we did not analyze the effect of medical comorbidity. A literature review shows that this remains a controversial issue with few studies documenting it. One study showed that a comorbidity scoring system did provide prognostic information while another study showed it did not.^42,43^ Third, we did not analyze the survival impact of smoking although heavy smoking was correlated with poor pathologic characteristics in adenocarcinoma.^44^ Fourth, we did not further analyzed the weight coefficient between different risk factors because of medium patient number. For disease-free survival, our result recommended that patient with more advanced stage would have high relapse rate and provide the clue of shorter surveillance interval and recommended precise image modality. Fifth, patients with disease relapse may receive different combinations of palliative therapy according to their conditions so that we could not differentiate the effect palliative therapy after disease relapse and its impact on overall survival. But we identified the overall survival was correlated to actual disease invasion status and tumor size and metastatic lymph node ratio were the dominant factors. Although limitations remain, our result is not only easily applicable for all resectable nonsmall cell lung cancer patients but also provides stratified disease-free survival information. In addition, our results also provide reference for the selection of an adequate surveillance imaging tool according to relapse risk stratification by poor prognostic factors.

## CONCLUSION

Poor prognostic factors correlated to disease-free survival were tumor size >5 cm, visceral pleural invasion, and patients needing to receive postoperative adjuvant therapy. Disease-free survival of resectable nonsmall cell lung cancer patients can be stratified by these 3 factors. In addition, disease relapse was correlated to the number of poor prognostic factors. Chest tomography may be recommended for patients with 1 or more poor disease-free survival risk factors, because more than half of these patients were identified with disease relapse.
